# Supplementary material for: Contrasting patterns of extrasynaptic NMDAR-GluN2B expression in macaque subgenual cingulate and dorsolateral prefrontal cortices
Source: Front Neuroanat. 2025 Apr 4;19:1553056. doi: 10.3389/fnana.2025.1553056 (PMC12006084; doi:10.3389/fnana.2025.1553056)
Supplement: Supplementary file 1 [file Data_Sheet_1.docx]

SUPPLEMENTARY FILE

**SUPPLEMENTAL FIGURE 1**

**
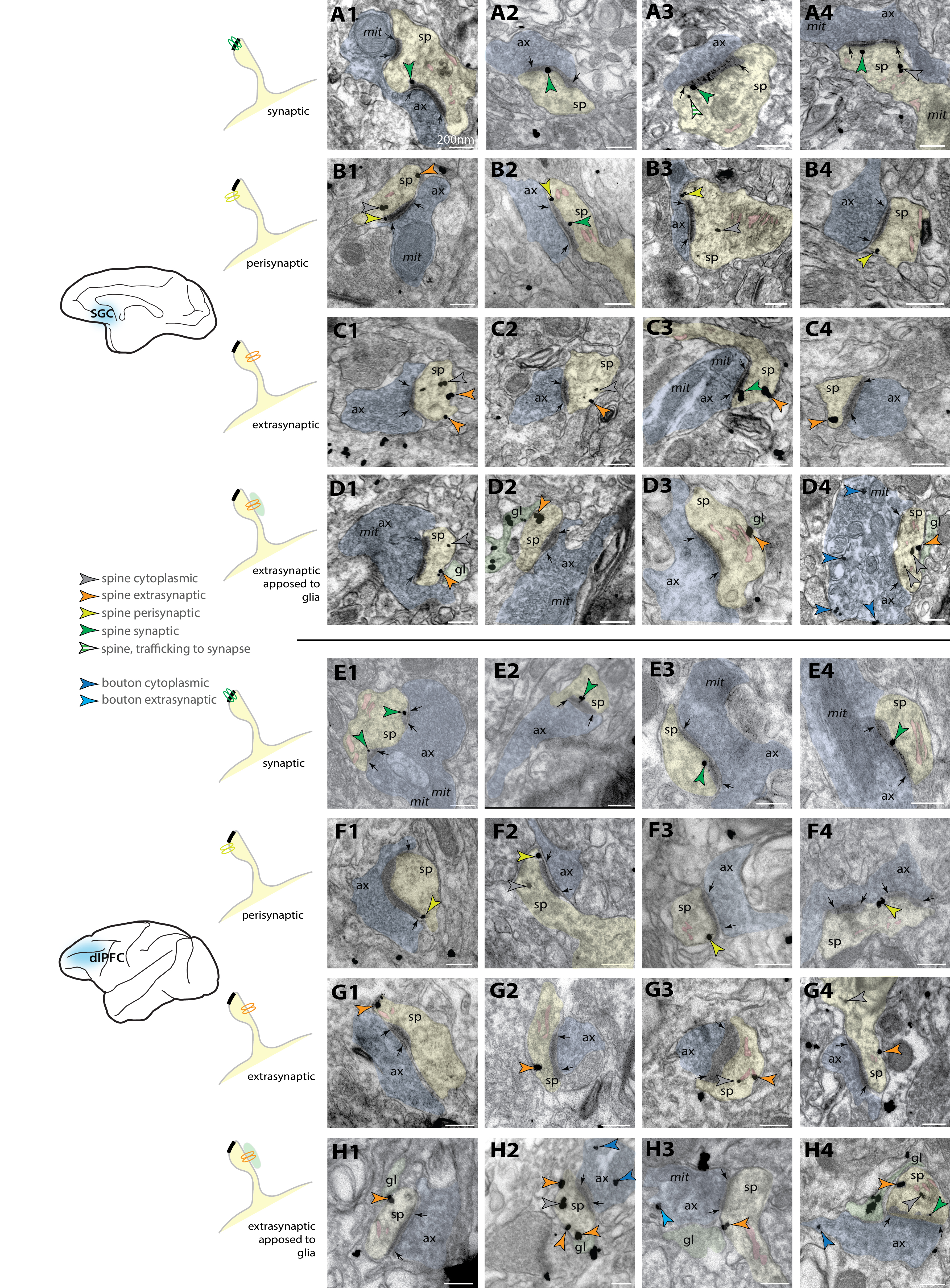
**

**Figure S1 – Additional examples of SGC and dlPFC NMDAR-GluN2B labeling in spines**

***A*,** Electron micrographs of SGC spines (pseudocolored yellow), receiving synapses (denoted by black arrows) formed by axon terminals (pseudocolored blue). NMDAR-GluN2B are prominent expressed in the synapse (green arrowheads), or at cytosolic locations (grey arrowheads). ***B***, SGC spines with prominent perisynaptic NMDAR-GluN2B (yellow-green arrowheads). NMDAR-GluN2B were classified as perisynaptic when within ~100nm of membrane distance from the synapse. ***C,*** SGC spines with NMDAR-GluN2B expressed in the extrasynaptic membrane (orange arrowheads). ***D*,** SGC spines with extrasynaptic NMDAR-GluN2B apposed to glial-like processes (pseudocolored green), which sometimes also express NMDAR-GluN2B (*e.g.*, **D2**). Presynaptic cytosolic NMDAR-GluN2B (blue arrowheads) are occasionally evident (*e.g.*, **D4,** blue arrowheads). ***E-H,*** same as above but for dlPFC spines. Scale bars, 200nm; ax, axon; mit, mitochondria; sp, spine

**SUPPLEMENTAL FIGURE 2**

**
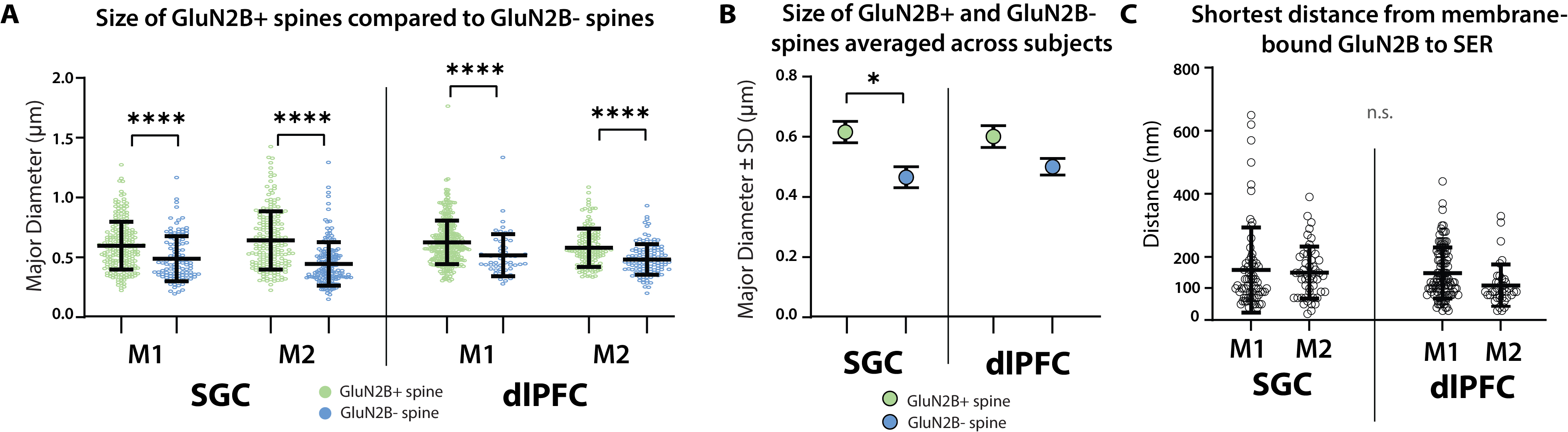
**

**Figure S2 – Quantitative characteristics of NMDAR-GluN2B+ spines and proximity of NMDAR-GluN2B to SER in spines**

***A,*** Swarm plot of the major Feret’s diameter of spines in SGC and dlPFC that were NMDAR-GluN2B+ (green) and GluN2B- (blue) in plane. Individual values are depicted as circles, and thick black lines depict the sample mean ± standard deviation (SD). Mann-Whitney U tests (two-tailed) were performed to test for differences between NMDAR-GluN2B+ and NMDAR-GluN2B- spines within each area and case (M1 SGC: U=8424, with 221 GluN2B+ spines and 113 GluN2B- spines, p<0.0001; M2 SGC: U=7905 with 192 GluN2B+ spines and 199 GluN2B- spines, p<0.0001; M1 dlPFC: U=5700 with 336 GluN2B+ spines and 56 GluN2B- spines, p<0.0001; M2 dlPFC: U = 5560 with 140 GluN2B+ spines and 124 GluN2B- spines, p<0.0001). ***B***, Feret’s spine diameter averaged across cases for each area for GluN2B+ (green) and GluN2B- spines (blue) (One-way ANOVA, F(3,4) = 12.66, p=0.02; post-hoc Tukey test SGC GluN2B+ vs. GluN2B-, p=0.0235) ***C,***  Swarm plot depicting the shortest distance from membrane bearing the NMDAR-GluN2B to the nearest spine apparatus SER membrane for membrane-bound NMDAR-GluN2B immunogold particles found in spines (M1 SGC n=68; M2 SGC n=49; M1 dlPFC n=109; M2 dlPFC n=38). Swarm plot also depicts mean and standard deviation (thick black lines). *, p < 0.05; ****, p<0.0001; n.s., not significant

**SUPPLEMENTAL FIGURE 3**

**
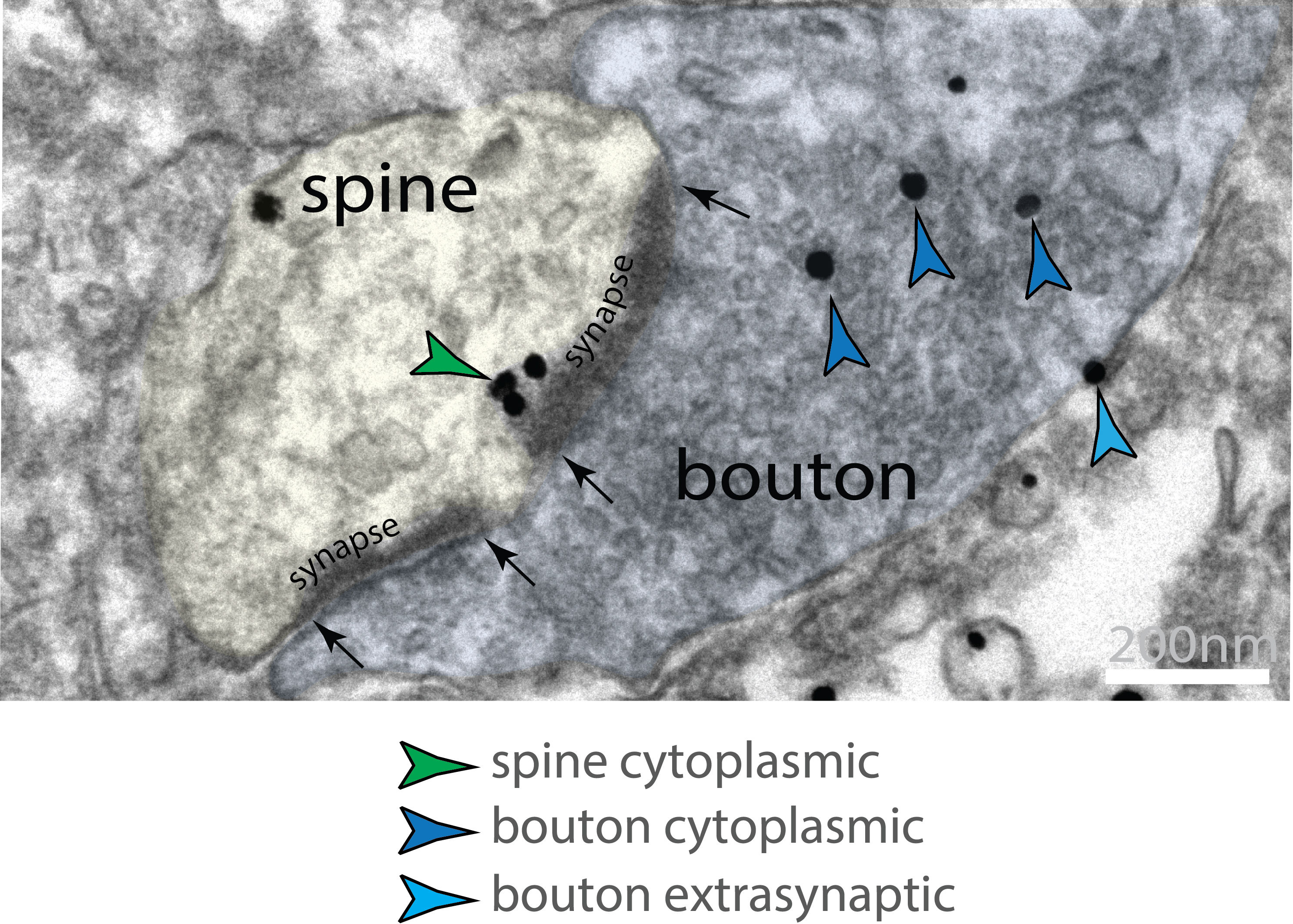
**

**Figure S3 – Presynaptic NMDAR-GluN2B**

An SGC spine (sp, pseudocolored yellow) with prominent NMAR-GluN2B synaptic labeling (green arrowhead), and presynaptic NMDAR-GluN2B in the bouton (ax, pseudocolored blue) in the cytosol (darker blue arrowheads) amidst the vesicles, or on the extrasynaptic bouton membrane (lighter blue arrowhead). Black arrows denote the boundaries of the perforated synapse.

**SUPPLEMENTAL FIGURE 4**


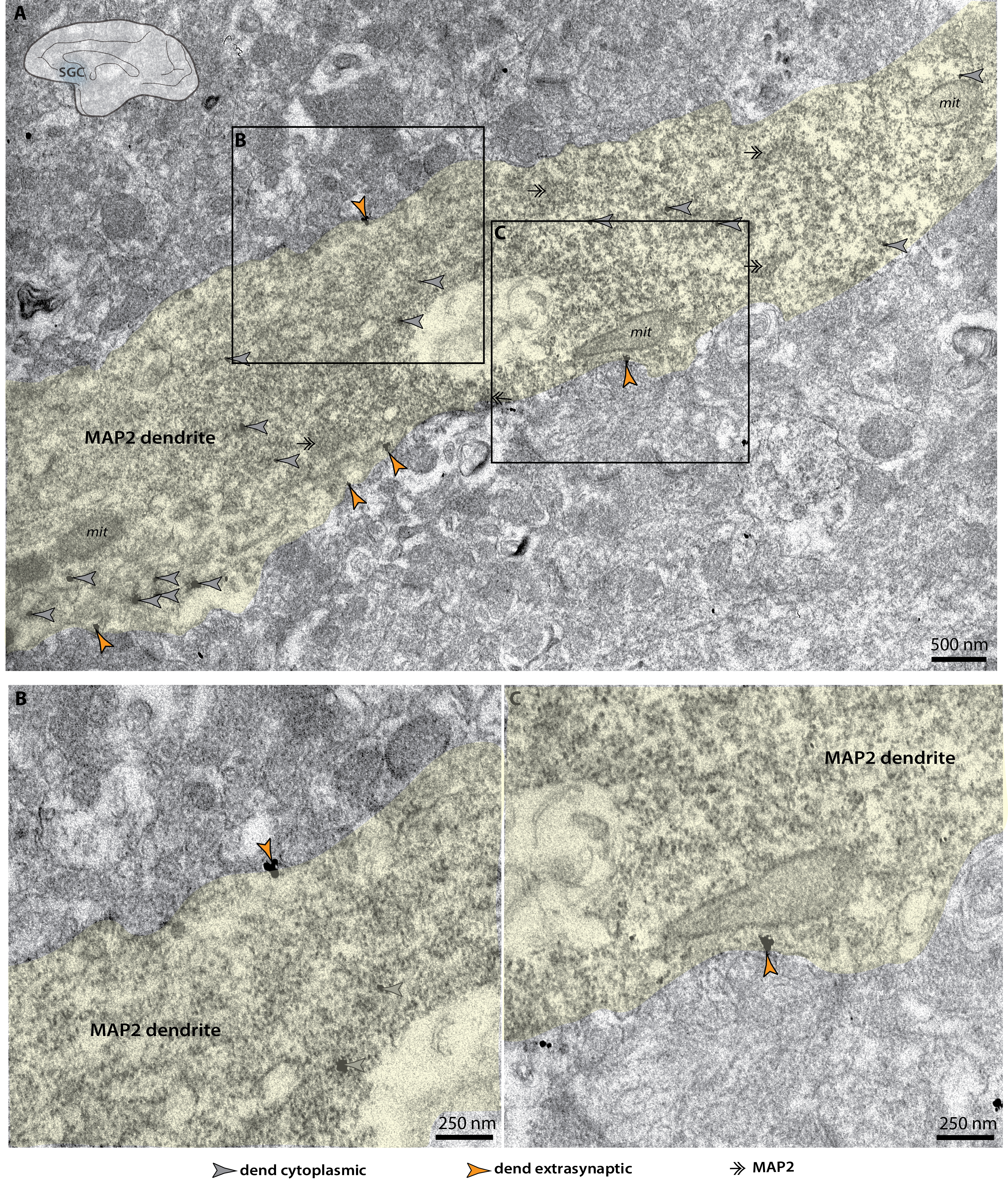


**Figure S4 – Extrasynaptic NMDAR-GluN2B in MAP2+ dendrite in SGC**

***A***, A MAP2+ putative excitatory dendrite (pseudocolored yellow) with NMDAR-GluN2B labeling at intracellular (grey arrowheads) and extrasynaptic (orange arrowheads) locations. ***B,C***, Insets from **A**. MAP2, microtubule-associated protein 2; mit, mitochondria

**SUPPLEMENTAL FIGURE 5**

**
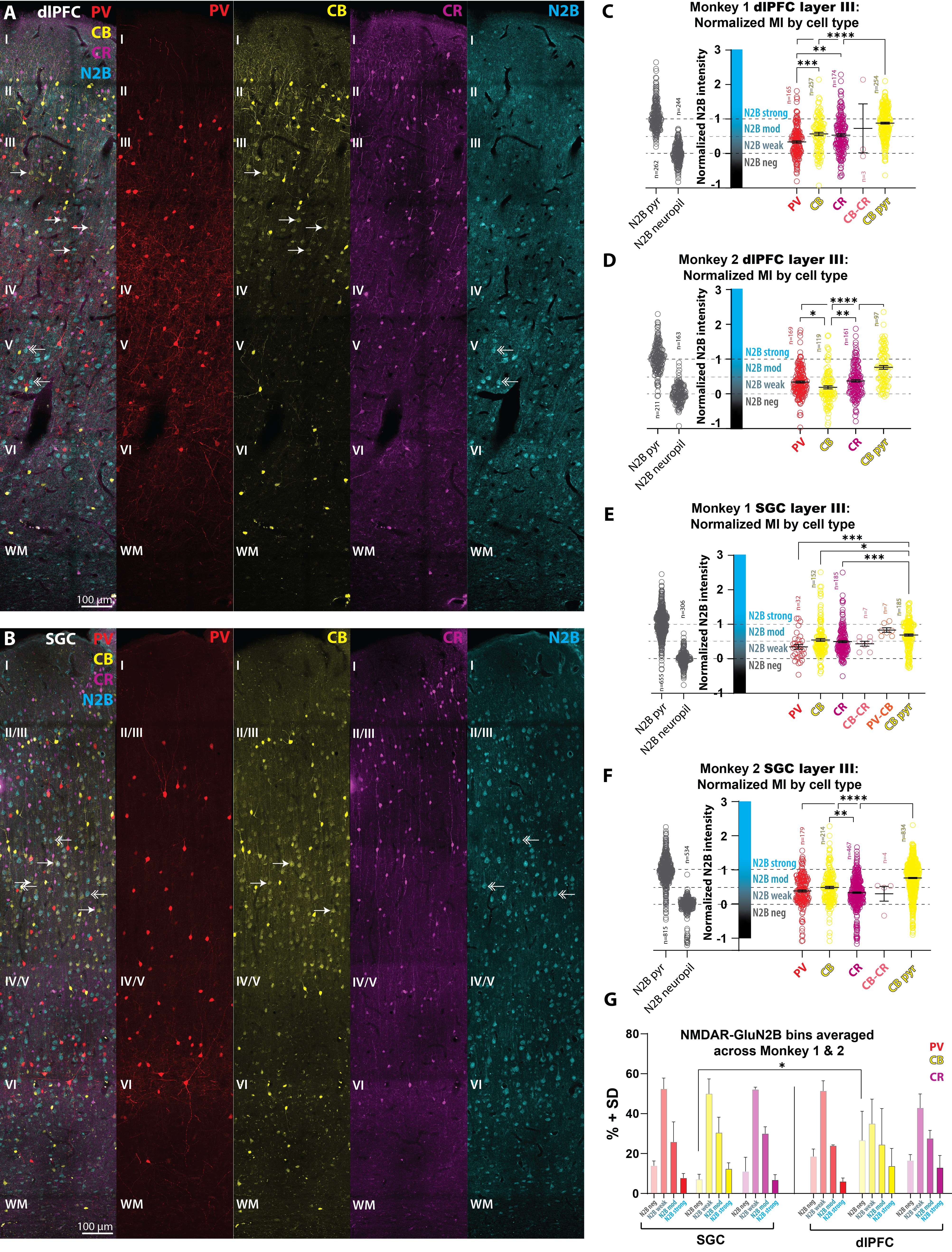
**

**Figure S5 –** **Supplemental MLIF depicting columnar expression of CBPs and NMDAR-GluN2B, as well as detailed analyses by subject for SGC and dlPFC layer III NMDAR-GluN2B expression across CBP types in Monkey 1 and Monkey 2.**

Tiled images obtained via confocal microscopy of immunolabeled PV (red), CB (yellow), CR (magenta), and NMAR-GluN2B (cyan) in SGC (**A**) and dlPFC (**B**) across all laminar compartments. Images were systematically sampled from layer III for all quantitative analysis depicted in ***C-G***. For each image, we isolated the NMDAR-GluN2B channel. Then we segmented i) NMDAR-GluN2B+ pyramidal-like neurons, as judged by morphology (large pyramidal-shaped soma, thick apical-like dendrite oriented toward layer I, examples depicted with double-headed arrows in cyan channel of panels **A-B**); and ii) manually selected immunonegative regions of tissue with no labeled NMDAR-GluN2B processes as a measure of immuno-”negative” labeling. We measured the mean intensity (MI) in all of these traces, and computed the mean for both categories (GluN2B+ pyramidal neurons or immunonegative background). We then used the other channels to segment the somata of PV, CB, and CR neurons, and measured the MI for each PV, CB, and CR neuron. Some layer III pyramidal neurons are lightly labeled for CB, as has been previously described (Kondo et al., 1999; Joyce et al., 2020). These have pyramidal-like morphology and faint CB labeling, which we called “CB-pyrs” (white arrows in CB panels in ***A-B***), as a separate population from the CB inhibitory neurons (“CB”). We then used the mean NMDAR-GluN2B expression of the pyramidal neurons and of the immunonegative “neuropil” regions to create a normalized index of expression for each inhibitory neuron, where 0 was the average across sampled neuropil regions, and 1 was the average across sampled pyramidal-like neurons. This index forms the y-axis for **C-F,** and the distributions and individual values of the sampled neurons and neuropil regions used to determine this axis is shown in the left half of each plot. Individual circles for each plot represent a cell, and these data were pooled across images after normalization. We divided the index into four equal bins from [0,1], delineated by dotted lines (Negative, at or below the average MI across sampled immunonegative regions; Weak; Moderate; or Strong, which was defined as at or above the average MI across sampled pyramidal neurons). Compiled data for Monkey 1 in SGC (**C**, One-way ANOVA, F(5,562)=6.314, p<0.0001, with post-hoc Tukey test) and dlPFC (**D** One-way ANOVA, F(4,701)=35.97, p<0.0001, with post-hoc Tukey test), and for Monkey 2 in SGC (**E,** One-way ANOVA, F(4,1693)=75.90, p<0.0001, with post-hoc Tukey test) and dlPFC (**F,** One-way ANOVA, F(3,542)=30.23, p<0.001, with post-hoc Tukey test)*.* Sample sizes are listed along with the distributions (“n=”) ***G,*** Mean percent across cases of CBP+ inhibitory neurons by type that fell into Negative, Weak, Moderate, or Strong bins (Three-way ANOVA, significant main effect for expression level F(3,24) = 63.302 p<0.001, η^2^=88.8; post-hoc Bonferroni pairwise comparisons revealed a significant difference for CB neurons in the N2B negative expression level category, p=0.012). CB, calbindin; CBP, calcium-binding protein; CR, calretinin; MI, mean intensity; PV, parvalbumin; WM, white matter. *, p < 0.05; **, p< 0.01, *** p< 0.001; ****, p<0.0001

**SUPPLEMENTAL FIGURE 6**

**
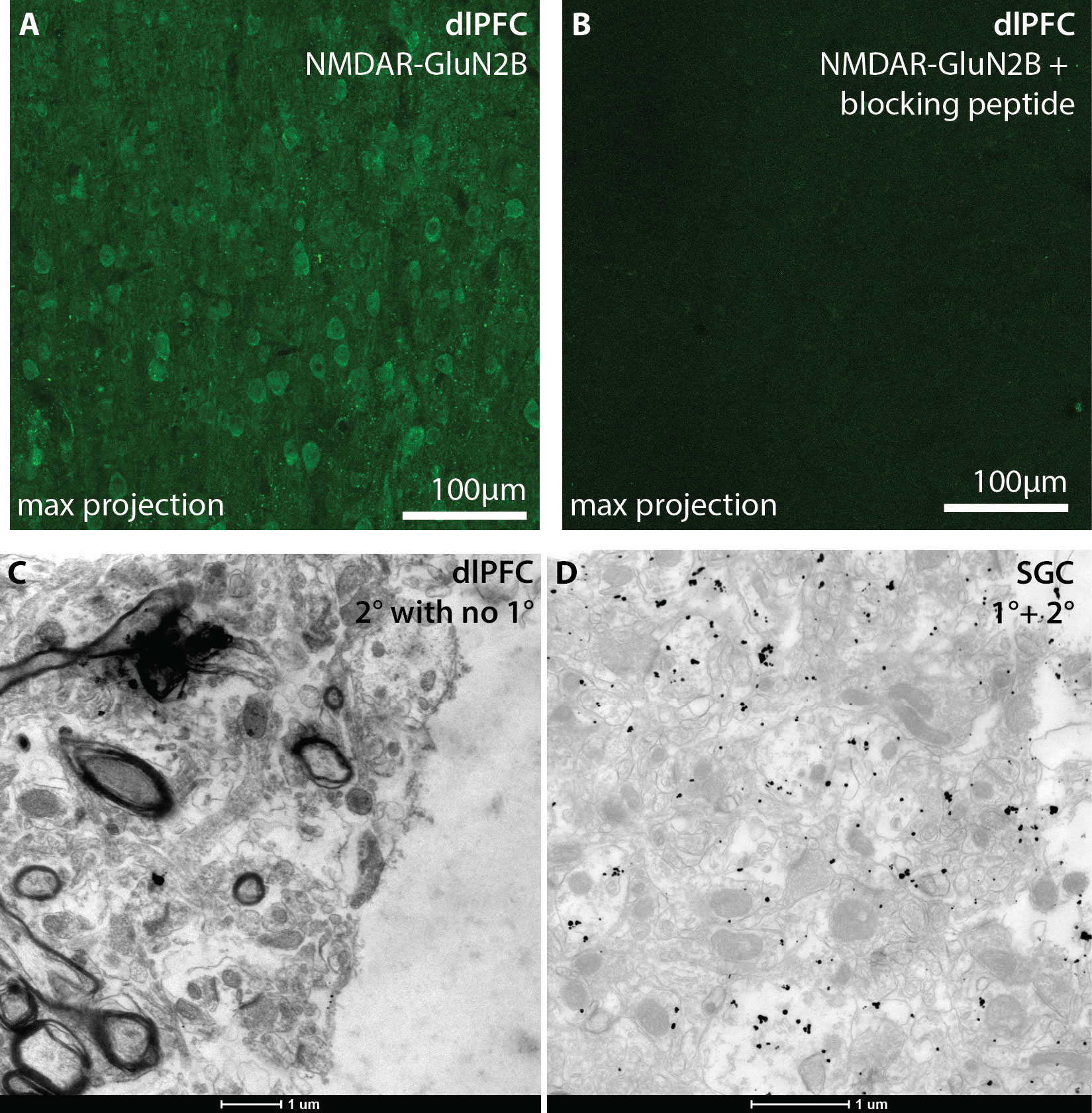
**

**Figure S6 – Immunohistochemical controls**

***A***, Confocal microscopy images depicting layer III dlPFC single-labeled for NMDAR-GluN2B using the Alomone rabbit anti-GluN2B (cat #AGC-003) antibody. ***B***, A control section incubated in a separate aliquot of primary antibody solution used in **A**, but with the addition of the Alomone blocking peptide (cat #BLP-GC003). The negligible labeling observed after imaging with the same parameters as **A** suggests that the non-paratope region of the antibody had negligible interactions in our tissue. ***C***, Electron micrograph from a control section of tissue with the omission of the primary antibody (1°) but all other procedures intact. The image was captured at the edge of the tissue “coming into plane”, where antibody penetration is at its greatest, often producing some noise or background level labeling. ***D***, Electron micrograph from the same batch of tissue, from a section treated with both the primary antibody and secondary antibody (2°), and all other procedures held constant. The image is taken near the edge of the tissue “coming into plane” (right), where antibody penetration is typically greatest, often producing some noise or background level labeling.
